# Implications of computer tomography measurement in the management of renal tumours

**DOI:** 10.1186/1471-2490-8-13

**Published:** 2008-11-04

**Authors:** Rahul Mistry, Ramaswamy Manikandan, Penny Williams, Joe Philip, Peter Littler, Christopher S Foster, Keith F Parsons

**Affiliations:** 1 Department of Urology, Royal Liverpool University Teaching Hospital NHS Trust, Liverpool, UK; 2 Department of Radiology, Royal Liverpool University Teaching Hospital NHS Trust, Liverpool, UK; 3 Department of Histopathology Royal Liverpool University Teaching Hospital NHS Trust, Liverpool, UK

## Abstract

**Background:**

To compare radiographic measurement and pathological measurement of renal tumours to see if there was a significant difference between the two as this may have implications in the management.

**Methods:**

We retrospectively analyzed CT measurements of 106 consecutive patients who underwent either radical or nephron sparing surgery in our institution and compared this to the actual measurement of the surgical specimen. The largest axial measurement was compared as this is the primary consideration before offering either treatment modality.

**Results:**

The mean age of the patients was 64 years (range 31–92). There were 76 males and 30 females. The median tumour size was 70 mm (range 16–175) on CT and 65 mm (range 15–90) on pathological measurement. 25 patients had a CT size ≤ 40 mm. CT tended to overestimate the size of tumours in 41 patients, underestimate in 45 and agree with surgical size in 20 patients. Statistically there was no significant difference between the two measurements (p = 0.7, Wilcoxon sign ranked test). When subdivided into tumours less than 40 mm (p = 0.7) and more than 40 mm (p = 0.09) again there was no statistically significant difference between the two measurements. However in 5(5%) patients who were not offered nephron sparing surgery based on CT findings (size > 40 mm) the pathological size was ≤ 40 mm (p = < 0.001, Fishers Exact test). Pathologically the tumours were classified as renal cell carcinoma (n = 98), angiomyolipoma (3), and oncocytoma (5).

**Conclusion:**

CT measurement of renal tumour size correlates well with the actual size of the tumour. However CT does tend to overestimate the size in a small number of patients which may have a bearing on the modality of treatment offered.

## Background

Radical nephrectomy is the standard treatment for localized renal cell carcinoma (RCC) in patients with a normal contra lateral kidney. However, in patients with a solitary functioning kidney, nephron sparing surgery (NSS) is mandatory [1]. NSS is also desirable in benign renal tumours such as angiomyolipoma [2]. It therefore should also be considered when there is uncertainty about the diagnosis because of the possibility of a benign renal tumour, angiomyolipoma or a complex cyst. In recent years, NSS has also been proposed for RCC with a normal contra-lateral kidney with excellent five year cancer-specific survival results being reported [3-5]. Such a surgical option is particularly suited for small RCCs, which seem to be best suited for renal sparing surgery [6]. In fact, with the advent and development of ultrasound and computerized tomography (CT), small RCCs are being detected with increasing frequency [7,8]. With this increase of number, there has been a decrease in the average size of tumours found [7,8]. The relationship between the clinical and pathologic size of renal tumours has been studied before with variable results [9-14]. Some of studies suggested preoperative CT imaging may overestimate the pathologic size, which may have implications for planning NSS [9-13]. Our aim was to determine what extent do CT measurements over or under-estimate the size of small renal tumours and how it can impact of their management.

## Methods

We retrospectively identified 106 consecutive patients who underwent a radial nephrectomy or NSS for a suspected renal tumour between the years 2000 and 2006. All patients underwent a contrast enhanced CT scan and pathological examination of the specimen at one institution. Data on the CT measurements were taken from the radiology reports. The CT device used and the grade of the reporting Radiologist was noted. The CT scans were reviewed by a team of Radiologists with a special interest in urology. Eighty eight percent (93) of the CT scans were reported by Consultant Radiologists and 12% (13) by Registrars. 48% (51) of tumours were scanned using a single slice helical scanner (GE CT Lxi™, GE Healthcare, Giles, UK) with the images viewed on 7 mm slices; 21% (22) with a single slice non helical scanner (GE Hispeed advantage™, GE Healthcare, Giles, UK) with images viewed on 5 mm slices and 31% (33) with a 16 slice helical scanner (Siemens Sensation™, Siemens Medical Solutions, Frimley, UK) with the images reconstructed and viewed on 1 mm slices.

The resected specimen was analysed by a single Urological Histopathogist. The tumour was orientated and transected as per the Pathological cut-up protocol with the macroscopic dimensions being measured using a ruler.

For both the CT scan and the surgical tumor specimen, the largest transaxial or transverse diameter, respectively, was used to measure the size of the tumor, using either ruler. One measurement of maximum tumor diameter was used, since it is equivalent to the bi-dimensional measurement to assess changes in solid tumors, even for non-spherical tumours.

**Figure 1:**
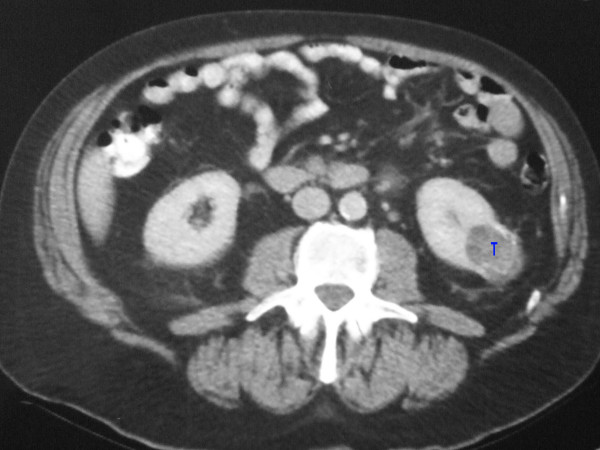
CT picture of a left lower pole renal tumour (T).

**Figure 2:**
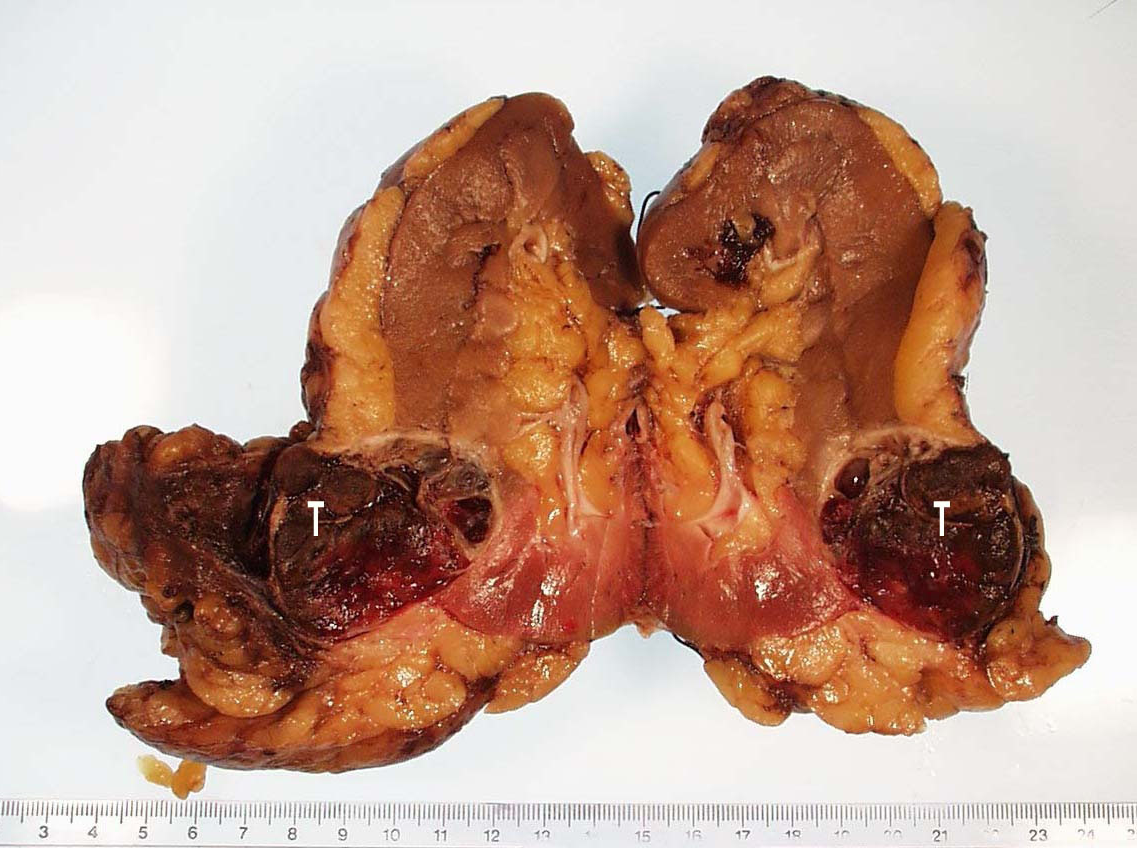
Same tumour after nephrectomy (T).

The largest axial dimension was recorded from the CT report. This was then compared to the actual measurement taken from the histopathological report. Figure 1 shows the CT picture of a left lower pole renal tumour and Figure 2 shows the resected specimen of the same tumour. Pathologically the tumours were classified as renal cell carcinoma, angiomyolipoma, and oncocytoma. These two comparable dimensions were analysed using the Wilcoxon sign ranked test and the Fishers Exact test.

## Results

106 patients were identified as having a radical nephrectomy or nephron sparing surgery. This included 76 males and 30 females with a mean age 62 years (range 31–92). Of the 106 tumours excised, 98 (92%) were classified as renal cell carcinoma, 3 (3%) angiomyolipoma, and 5 (5%) oncocytoma.

The median tumour size was 70 mm (range 16–175) on CT and 65 mm (range 15–90) on pathological measurement. 22 patients had a CT size ≤ 40 mm. CT tended to overestimate the size of tumours in 41 patients, underestimate in 45 and agree with surgical size in 20 patients. Statistically there was no significant difference between the two measurements (p = 0.7, Wilcoxon sign ranked test). When subdivided into tumours less than 40 mm (p = 0.7) and more than 40 mm (p = 0.09) again there was no statistically significant difference between the two measurements. However in 5 (5%) patients who were not offered nephron sparing surgery based on CT findings (size > 40 mm) the pathological size was ≤ 40 mm (p = < 0.001, Fishers Exact test). When further subdivided into various size ranges the difference between CT and pathology are shown in Table 1 and 2. In general CT tended to overestimate tumours less than 70 mm and underestimate tumours greater than 70 mm.

**Table 1:** Comparison between pathological and CT size

Pathologic Size in mm	Number of patients	Mean CT size (mm)	Pathological size (mm)	Difference	% Difference	p value (Wilcoxon sign Ranked Test)
10 to < 20	2	27	15.5	12.5	80	0.18
20 to < 30	6	25.5	25	0.5	2	0.09
30 to < 40	9	41	31.9	9.1	28	0.23
40 to < 50	11	44.5	42.3	2.2	5.2	0.6
50 to < 60	10	47.2	53.3	-6.1	-11	0.02
60 to < 70	20	69.3	61.8	7.5	12	0.05
≥ 70	48	95	101.8	-6.8	-6.6	0.06

Total	106	49.9	47.37	2.53	5.3	

**Table 2:** Comparison between CT and Pathology when Tumours are stratified according to the TNM criteria

Pathological Stage (size in mm)	Number of Patients	Mean CT size (mm)	Mean Pathological size (mm)	Difference (mm)	Difference %	p value
T1a/T3a (< 40 mm)	17	33.9	27.5	6.4	23	0.13
T1b/T3a (40–70 mm)	47	58.6	56.4	2.2	3.9	0.48

T2/T3a (> 70 mm)	42	99	106	-7	-6.6	0.07

## Discussion

The importance of the size of renal tumours when considering NSS is increasingly becoming more apparent. Since the numbers of incidentally discovered tumours are up to 25–40% in developed countries, there is an increasing demand for NSS[8]. In fact these patients are being detected incidentally during routine health care screening programs, whereas historically most cases were diagnosed following investigations for flank pain or hematuria[15]. The rising trend that has been observed is primarily due to enhanced detection by widely used non-invasive imaging techniques, such as ultrasonography and computer tomography (CT) [16]. These incidentally discovered RCCs have tended to be smaller and of a low pathological stage than those detected in symptomatic patients. Improved survival has been demonstrated in these patients, compared with those with symptomatic tumour presentations [4,17].

Patients who have incidental renal tumours less than 40 mm cm treated with NSS have been shown to have a better outcome than those with tumours greater than 40 mm, with the 5 year disease-free survival rate being 95–100%[4,18]. Whilst this study used the pathologic size in determining the appropriate size cut-off for NSS, it is the radiographic size that is used in routine practice when considering NSS. However the use of 40 mm as a rigid cut off point is changing as Patard *et al *[19] have shown no difference in the cancer specific death rates in patients undergoing radical or partial nephrectomy for tumours between 40 mm and 70 mm (9% vs.6.2%, p < 0.6).

While there is increased detection of these small renal masses, an accurate pre-operative diagnosis can often be difficult with inter-radiologist variation in CT reporting techniques. Some CT scanners allow 3-dimensional reconstruction with the aid of a multi-slice scanners (e.g. Siemens 16 slice Sensation helical scanner), whilst others simply allow only a 2-dimensional measurement with an estimate of the 3rd dimension on single slice machine. Punnen et al assessed 29 renal masses measured by three radiologists in three different planes: axial, perpendicular and superior-inferior obtained using a 4-slice, multirow helical CT scanner [20]. The inter-reader and intra-reader variability in the size of measurements found the highest variance between and within readers was in the superior-inferior axis. This axis was displayed from reconstructed images, making it more difficult to define the edges of the mass along that dimension. The least inter-observer and intra-observer variability was noted in the axial diameter; however there was only one tumour between 3 and 4 cm included in this study.

According to Steinbach and associates [2], the specificity of the CT scan in the diagnosis of small renal tumours is between 85% and 90%; they also noted that the differentiation of oncocytoma, adenomas and haemorrhagic cysts could be problematic. Jamis-Dow *et al*[10] noted that for 10–35 mm lesions, 80% and 82% were correctly characterized with CT and ultrasound, respectively (n = 205).

Several studies have examined the relationship between the radiographic and pathologic tumour size [9-13]. Schlomer *et al *[13] retrospectively identified 126 patients who underwent a nephrectomy or NSS with in 60 days of CT scanning and found that the scans tended to overestimate the size of pT1a (p = 0.009) and pT1b tumours (p = 0.087) and underestimate tumours > 70 mm. Our study concurs with their findings (Table 2). Herr [9] looked at 50 patients who underwent NSS who had CT scans within 4 weeks of the surgery. They found that tumours were a mean of 6.3 mm smaller than the CT scans stated. This was particularly noticeable in the tumours ≥ 35 mm, with a mean decrease of 9.5 mm (n = 17). This difference was attributed to the shrinkage of the tumours by temporary renal artery occlusion and surface hypothermia. In another study by the same author, of the 87 incidentally identified renal tumours, clear cell carcinomas decreased on average of 9.7 mm (p = 0.0001) versus 39 mm (p = 0.0001) for the other tumours[11]. They also found that tumours > 30 mm, there was a change of 8.7 mm (p = 0.001) when compared to the pathological size. Irani *et al *[12] analysed 100 patients who underwent radical nephrectomies and found that CT estimate and surgical measurement of tumour were highly correlated (r = 0.9; p < 0.001). The median tumour size on CT was 70 mm compared to 60 mm on pathological sampling, with a significant difference (p = 0.005). Yaycioglu *et al *[14] retrospectively looked at 291 nephrectomies and found a high correlation between the clinical and pathological size (p < 0.0001).

Patients in our institution were offered NSS if the maximal diameter of the renal tumour was less than 40 mm on the CT scan as per protocol.

## Conclusion

Our retrospective study supports the findings of previous studies with 41 (39%) patients having an overestimation of the size (ns). However, in 5 (5%) patients who were not offered NSS based on CT findings (size > 40 mm) the pathological size was ≤ 40 mm (p < 0.001, Fishers Exact test). This may therefore have had a bearing on the treatment options offered to the patients. Accurate CT measurements of renal tumours play a vital role in deciding whether to proceed with NSS or not in this group of patients, therefore our protocol is under revision to incorporate this variance in CT interpretation as it may affect the surgical decision making.

Since our data was retrospectively reviewed, the results are subject to an observational variability. Our discrepancies might have been caused by the lack of standardization in the radiologic and pathologic measurement techniques. As well as the use of three different radiographic imaging equipment, the tumours maximal dimensions may well have not been measured in the same geometric plane therefore producing errors in comparison. Ideally in the future, a single multi-slice helical scanner should be used with a single radiologist reporting. Our study can be improved with a power calculation as the number patients and tumours were small. This study should be followed up by a multicentre prospective study with preset protocols for assessing the CT and Pathological measurements.

## Competing interests

The authors declare that they have no competing interests.

## Authors' contributions

RM – Drafted the manuscript. RMK – conceived the idea of the study and also corrected the manuscript. JP – helped with the manuscript and the overall data. PL – looked at the radiological data and protocol. CSF – reported on the histopathology and the pathological measurements data and KFP is the senior author and corrected the manuscript.

## Pre-publication history

The pre-publication history for this paper can be accessed here:



## References

[B1] Blute ML, Malek RS, Segura JW (1988). Angiomyolipoma: Clinical metamorphosis and concept for management. J Urol.

[B2] Steinbach F, Stockle M, Muller SC, Thüroff JW, Melchior SW, Stein R, Hohenfellner R (1992). Conservative surgery of renal cell tumours in 140 patients: 21 years of experience. J Urol.

[B3] Morgan WR, Zincke H (1990). Progression and survival after renal-conserving surgery for renal cell carcinoma: Experience in 104 patients and extended follow-up. J Urol.

[B4] Licht MR, Novick AC, Goormastic M (1994). Nephron sparing surgery in incidental versus suspected renal cell carcinoma. J Urol.

[B5] Novick AC (1995). Partial nephrectomy for renal cell carcinoma. Urol.

[B6] Steinbach F, Stockle M, Hohenfellner R (1995). Current controversies in nephron-sparing surgery for renal-cell carcinoma. World J Urol.

[B7] Smith SJ, Bosniak MA, Megibow AJ, Hulnick DH, Horii SC, Raghavendra BN (1989). Renal cell carcinoma: earlier discovery and increased detection. Radiology.

[B8] Jayson M, Sanders H (1998). Increased incidence of serendipitously discovered renal cell carcinoma. Urology.

[B9] Herr HW (2000). Radiographic vs surgical size of renal tumours after partial nephrectomy. BJU Int.

[B10] Jamis-Dow CA, Choyke PL, Jennings SB, Linehan WM, Thakore KN, Walther MM (1996). Small (< or = 3-cm) renal masses: detection with CT versus US and pathologic correlation. Radiology.

[B11] Herr HW, Lee CT, Sharma S, Hilton S (2001). Radiographic versus pathologic size of renal tumours: implications for partial nephrectomy. Urology.

[B12] Irani J, Humbert M, Lecocq B, Pires C, Lefèbvre O, Doré B (2001). Renal tumour size: comparison between computed tomography and surgical measurements. Eur Urol.

[B13] Schlomer B, Figenshau RS, Yan Y, Bhayani SB (2006). How does the radiographic size of a renal mass compare with the pathologic size?. Urology.

[B14] Yaycioglu O, Rutman MP, Balasubramaniam M, Peters KM, Gonzalez JA (2002). Clinical and pathologic tumour size in renal cell carcinoma; difference, correlation, and analysis of the influencing factors. Urology.

[B15] Luciani LG, Cestari R, Tallarigo C (2000). Incidental renal cell carcinoma-age and stage characterization and clinical implications: study of 1092 patients (1982–1997). Urology.

[B16] Pantuck AJ, Zisman A, Belldegrun AS (2001). The changing natural history of renal cell carcinoma. J Urol.

[B17] Patard JJ, Rodriguez A, Rioux-Leclercq N, Guillé F, Lobel B (2002). Prognostic significance of the mode of detection in renal tumours. BJU Int.

[B18] Katz J, Shi W, Thaler HT, Reuter VE (2000). Surgical management of renal tumors 4 cm or less in a contemporary cohort. J Urol.

[B19] Patard JJ, Shvarts O, Lam JS (2004). Safety and efficacy of partial nephrectomy for all T1 tumours based on an international multicenter experience. J Urol.

[B20] Punnen S, Haider MA, Lockwood G, Moulding F, O'Malley ME, Jewett MA (2006). Variability in size measurement of renal masses smaller than 4 cm on computerized tomography. J Urol.

